# Effect of SGLT2-Inhibitors on Epicardial Adipose Tissue: A Meta-Analysis

**DOI:** 10.3390/cells10082150

**Published:** 2021-08-20

**Authors:** Walter Masson, Augusto Lavalle-Cobo, Juan Patricio Nogueira

**Affiliations:** 1 Council of Epidemiology and Cardiovascular Prevention, Argentine Society of Cardiology, Buenos Aires 1115, Argentina; walter.masson@hospitalitaliano.org.ar (W.M.); augustolavalle@gmail.com (A.L.-C.); 2 Endocrinology, Nutrition and Metabolism Investigation Center, Faculty of Health Science, National University of Formosa, Formosa 3600, Argentina

**Keywords:** epicardial adipose tissue, meta-analysis, sodium–glucose co-transporter-2 inhibitors

## Abstract

(1) Sodium–glucose cotransporter-2 inhibitors (SGLT2-i) reduce adipose tissue and cardiovascular events in patients with type 2 diabetes (T2D). Accumulation of epicardial adipose tissue (EAT) is associated with increased cardio-metabolic risks and obstructive coronary disease events in patients with T2D. (2) We performed a systematic review and meta-analysis of SGLT2-i therapy on T2D patients, reporting data on changes in EAT after searching the PubMed/MEDLINE, Embase, Science Direct, Scopus, Google Scholar, and Cochrane databases. A random effects or fixed effects model meta-analysis was then applied. (3) Results: A total of three studies (*n* = 64 patients with SGLT2-i, *n* = 62 with standard therapy) were included in the final analysis. SGLT2 inhibitors reduced EAT (SMD: −0.82 (−1.49; −0.15); *p* < 0.0001). An exploratory analysis showed that HbA1c was significantly reduced with SGLT2-i use, while body mass index was not significantly reduced with this drug. (4) Conclusions: This meta-analysis suggests that the amount of EAT is significantly reduced in T2D patients with SGLT2-i treatment.

## 1. Introduction

A close link exists between type 2 diabetes (T2D) and cardiovascular disease. Cardiovascular disease is the most prevalent cause of morbidity and mortality among people with type 2 diabetes [1].

There is growing evidence concerning the importance of epicardial adiposity in cardiometabolic risk. Epicardial adipose tissue (EAT) is composed of the adipose tissue depot immediately adjacent to the myocardium. Studies suggest that excessive EAT plays an important role in the genesis of coronary heart disease through potential paracrine or endocrine mechanisms by exerting inflammatory mediators, such as TNF-alpha, IL-6, adipocytokines, and leptin, which predispose to atherosclerosis [2,3].

Studies have shown that EAT is closely related to the extent and severity of coronary artery disease [4]. It is also associated with high-risk atherosclerotic plaque composition [5].

It is established that sodium–glucose cotransporter-2 inhibitors (SGLT2-i) provide effective glycemic control and have been shown to reduce atherosclerotic events, hospitalizations for heart failure, cardiovascular mortality, and the progression of chronic kidney disease. Large randomized controlled trials of SGLT2-i, empagliflozin (EMPA-REG OUTCOME), canagliflozin (CANVAS PROGRAM), and dapagliflozin (DECLARE-TIMI 58) have proven their efficacy in cardiovascular outcome in patients with T2D and high cardiovascular risk or established cardiovascular disease [6,7,8]. The effects of SGLT2-i expand far beyond these indications, and its use has been studied in the treatment of heart failure and chronic kidney disease, even in patients without diabetes [9]. The protective effects of SGLT2-i on the kidney are believed to be mediated by a number of both hemodynamic and nonhemodynamic mechanisms: reduction in blood pressure and vascular stiffness [10], restoration of the tubuloglomerular feedback [11], reduction in workload regarding ATP production [12], anti-inflammatory and antifibrotic effects, and reduction in oxidative stress [13].

In addition, these drugs are associated with body weight loss and can reduce abdominal visceral adipose tissue (VAT) [14].

Interestingly, the heart does not express SGLT2, but it is expressed in EAT [15]. Similarly, SGLT2-i increases glucose uptake, reduces the secretion of proinflammatory chemokines, and improves the differentiation of epicardial adipose tissue cells [15]. These data suggest a new potentially protective pathway for this group of drugs in cardiovascular disease. In other words, targeted pharmaceutical interventions with these antidiabetic drugs could help to return EAT to its physiological role.

Previously published studies have evaluated the association between SGLT2-i and EAT changes in T2D patients, although the results were conflicting [16,17,18,19,20,21,22,23]. To date, there are no published meta-analyses that have evaluated this topic.

Therefore, the main objective of the present meta-analysis is to assess the effect of SGLT2-i on changes in EAT in patients with T2D.

## 2. Materials and Methods

Data extraction and quality assessment: A systematic review was conducted based on the guidelines outlined in the Preferred Reporting Items for Systematic Reviews and Meta-Analyses (PRISMA) checklist [15]. Using the PICOS model, (patient, intervention, control, outcome, study), inclusion and exclusion criteria were created. The Meta-analysis Of Observational Studies (MOOSE) and the Strengthening the Reporting of Observational Studies in Epidemiology (STROBE) guidelines were used to check the reporting of observational studies [24,25]. A literature search was performed that identified studies of SGLT2-i published between January 2010 and June 2021 in English. Two independent reviewers searched the electronic PubMed/MEDLINE, Scielo, Google Scholar, Embase, and Cochrane Controlled Trials databases using the terms “Empagliflozin OR Dapagliflozin OR Canagliflozin OR SGLT2 inhibitor” AND “epicardial fat tissue OR epicardial adipose tissue OR subepicardial adipose tissue OR subepicardial fat tissue”.

The following inclusion criteria were used to select eligible studies: (1) randomized controlled trials (RCT) or observational studies with a cohort (prospective or retrospective) design, (2) studies that included patients with T2D, (3) studies that evaluated the relationship between SGLT2 inhibitor use and changes in EAT, and (4) follow-up duration ≥ 12 weeks. Exclusion criteria included duplicate studies, abstracts from unpublished studies, reviews, cross-sectional studies, case reports, and letters.

The primary endpoint was the change in EAT measured from baseline to follow-up, comparing groups of subjects on SGLT2 inhibitors alone versus standard therapy. For EAT measurements, computed tomography or magnetic resonance images were assessed.

The secondary endpoints were (a) change in lipids values between baseline and follow-up, (b) change in HbA1c levels values between baseline and follow-up, and (c) change in body mass index (BMI) between baseline and follow up. The quality of the included studies was assessed by two independent review authors using the Newcastle–Ottawa Scale (NOS) [26]. The NOS risk of bias assessment tool consists of nine items, which are divided into three domains (selection, comparability, and results). We considered that the studies showed a low, moderate, or high quality when the obtained score was 1 to 4, 5 to 6, or equal to or more than 7 points, respectively. Any discrepancy between the two reviewers was solved through discussion and by involving a third reviewer.

EAT thickness can be measured by an echocardiographic epicardial fat thickness test. Between the outer wall of the myocardium and the visceral layer of the pericardium, EAT is measured as the echo-free space. However, higher reproducibility and interobserver agreement have been shown by volumetric measurement [27]. EAT thickness is a parameter of surface that is used in qualitative analysis.

Epicardial fat volume (EFV) can be measured either by magnetic resonance imaging (MRI) or by computed tomography (CT). CT allows accurate measurement of epicardial and thoracic fat distances and volumes. Volumetric measurement has shown higher reproducibility and interobserver agreement (correlation coefficient 0.96) compared to distance measurements (0.58 for average epicardial fat volume) [28].

Statistical analysis: The summary effect of SGLT2-i on the primary endpoint was estimated. Measures of effect size were expressed as standardized mean difference (SMD), and the I^2^ statistic was calculated to quantify between trial heterogeneity and inconsistency. Depending on the value of I^2^, a fixed effects model (I^2^ < 40%) or a random effects model (I^2^ > 40%) was chosen. To compare mean effects between subgroups, a Z test was used.

Statistical analyses were performed using the R software (“The R Foundation for Statistical Computing”, Vienna, Austria) for statistical computing version 3.5.1 with additional specific packages [29]. A two-tailed *p*-value < 0.05 was considered statistically significant.

Analysis of publication bias: the tests for funnel plot asymmetry were not made because our research included only a small number of studies. In this context, the power of the tests was too low to distinguish chance from real asymmetry.

## 3. Results

The search included 107 potentially relevant articles after title screening, and 84 studies were excluded after abstract screening, as these were non-human or preclinical studies, or did not assess the treatment of interest. After full textual analysis, 12 studies were removed, as these did not include treatment or outcomes relevant to our study.

Seven eligible studies were identified and considered eligible for the qualitative analyses. Three studies examined the effect of SGLT2-i on EAT compared to a control group and were considered eligible for the quantitative analyses. A total of 64 subjects were allocated to receive SGLT2 inhibitors, while 62 subjects were allocated to the respective control arms.

**Figure 1:**
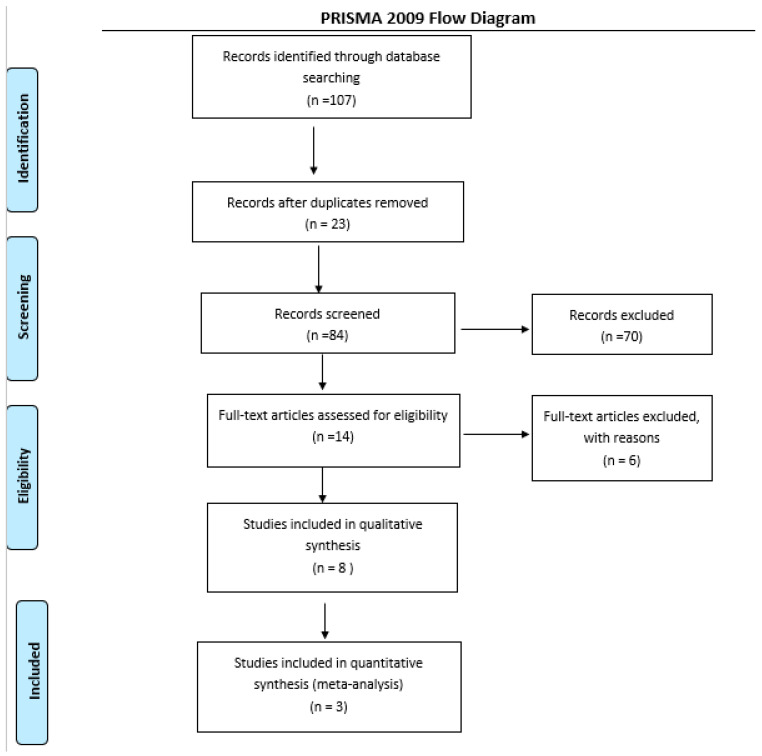
Flow diagram of the study screening process.

From the total of studies included in the systematic review, those that reported volume changes in EAT related to the use of SGLT2-i were selected for meta-analysis (computed tomography or magnetic resonance images). Three studies were not included in the quantitative analysis because they analyzed the changes in EAT through thickness, and two studies were not included because they were paired studies without a control arm. A flow diagram of the study’s screening process is shown in Figure 1.

**Figure 2:**
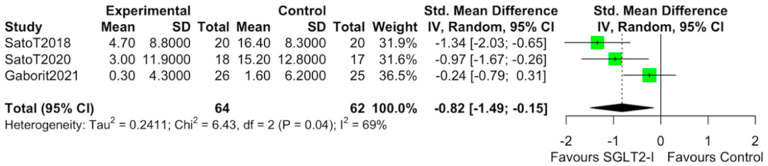
Effect of SGLT2 inhibitor therapy on epicardial adipose tissue (EAT). Random−effects, standardized mean difference (SMD), 95% confidence intervals (CI), and I^2^ statistics.

**Figure 3:**
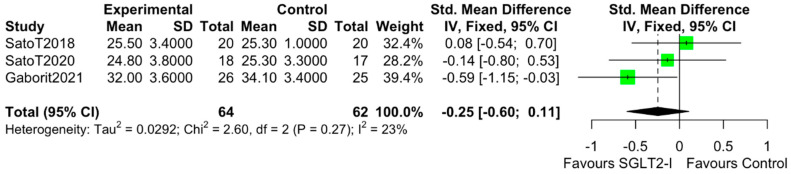
Effect of SGLT2 inhibitor therapy on body mass index (BMI). Fixed−effects, standardized mean difference (SMD), 95% confidence intervals (CI), and I^2^ statistics.

**Figure 4:**
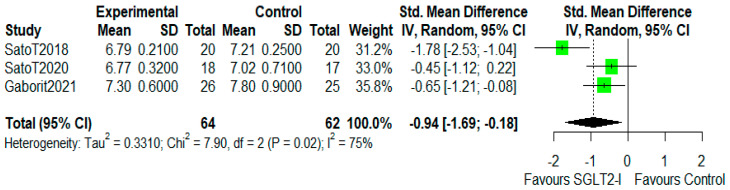
Effect of SGLT2 inhibitor therapy on HbA1c. Random−effects, standardized mean difference (SMD), 95% confidence intervals (CI), and I^2^ statistics.

**Figure 5:**
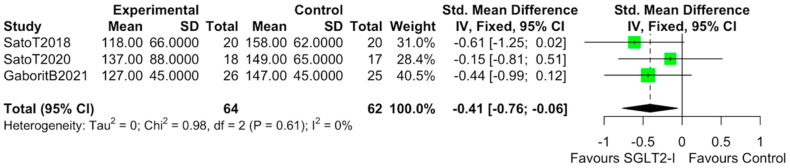
Effect of SGLT2-i therapy on TG. Fixed−effects, standardized mean difference (SMD), 95% confidence intervals (CI), and I^2^ statistics.

**Figure 6:**
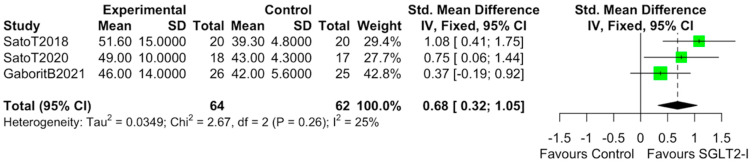
Effect of SGLT2-i therapy on HDL. Fixed−effects, standardized mean difference (SMD), 95% confidence intervals (CI), and I^2^ statistics.

**Figure 7:**
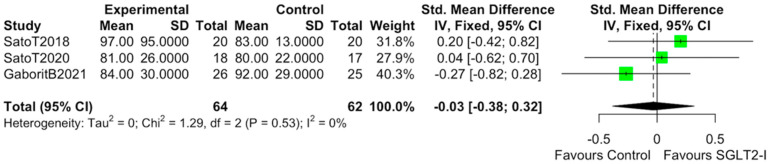
Effect of SGLT2-i therapy on LDL. Fixed−effects, standardized mean difference (SMD), 95% confidence intervals (CI), and I^2^ statistics.

The characteristics of the studies selected for our analysis are summarized in Table 1. The overall bias determined in all studies included in this systematic review was found to be low, indicating high-quality studies (Table 2). The quantitative analysis of the studies that included a control group showed that SGLT2-i use was associated with a reduction in the volume of EAT (SMD: −0.82 (−1.49 to −0.15); *p* < 0.0001) (Figure 2). Statistical heterogeneity was high (I^2^ = 69%). BMI levels were reported in all studies. After SGLT2-i, BMI levels were reduced but not significantly in comparison with the control group (SMD −0.25 (−0.60 to 0.11), *p* = 0.24) (Figure 3). The heterogeneity detected among the studies was low (I^2^ = 23%). HbA1c levels were reported in all studies. In comparison with the control arm, SGLT2-i was associated with a significant reduction in HbA1c levels (SMD: −0.93 (−1.69 to −0.18), *p* < 0.0001) (Figure 4). In addition, statistical heterogeneity was high (I^2^ = 74.7%). Plasma triglyceride (TG) levels were reported in all studies. SGLT2-i reduced TG levels significantly compared to the control arm (SMD −0.4103 (−0.7643; −0.0562), *p* = 0.0231) (Figure 5). There was low heterogeneity detected among the studies (I^2^ = 0%, *p* = 0.61). High-density lipoprotein (HDL) levels were reported in all studies. SGLT2-i increased HDL levels significantly compared to the control arm (SMD −0.6848 (0.3221; 1.0474), *p* = 0.0002) (Figure 6). There was low heterogeneity detected among the studies (I^2^ = 25%, *p* = 0.26). Low-density lipoprotein (LDL) levels were reported in all studies. SGLT2-i did not change LDL levels compared to the control arm (SMD −0.0320 (−0.3823; 0.3183), *p* = 0.85) (Figure 7). There was low heterogeneity detected among the studies (I^2^ = 0%, *p* = 0.52).

## 4. Discussion

In this systematic review, the main observational studies that evaluated the impact of SGLT2-i on EAT in patients with T2D were analyzed. In addition, in this meta-analysis, the use of SGLT2-i compared with the control arm was associated with a marked reduction in the EAT volume.

Abnormally accumulated visceral fat favors insulin resistance. This can lead to a reduction in insulin sensitivity, increase the secretion of proinflammatory cytokines in adipose tissue, and promote the development of cardiovascular diseases [30,31]. EAT is one of the visceral fat stores in the body, and some authors have suggested that measurement of EAT is a substitute for that of VAT [32]. A previously published meta-analysis suggests that the amount of EAT is significantly higher in T2D patients than in non-T2D patients [33]. Therefore, interventions designed for metabolic control in the patient with T2D could have a beneficial impact on EAT [27].

However, the results published to date are conflicting. Zsóri et al. reported that in new-onset T2D, metformin reduced fat deposits in the liver without an effect on the pericardium [34]. In addition, another report showed that pioglitazone, but not metformin, increased pericardial fat volume in T2D patients [35]. Conversely, some studies showed that liraglutide (an analog of glucagon-like peptide-1) and sitagliptin (a dipeptidyl peptidase-4 inhibitor), caused a substantial and rapid EAT reduction [36,37]. With the meta-analysis technique, our study analyzed all of the available evidence for the first time, exploring the effect of SGLT2 inhibitors on EAT.

In Gaborit et al. 2021, empagliflozin treatment did not reduce EAT, despite the reduction in BMI, Hba1c, and VAT. One possible mechanism when we compared Sato T et al. 2020 [17] and Sato T et al. 2018 [16] was the difference in body compositions between Asians and Europeans. Sato T et al. used CT imaging, and the follow-up occurred after 24 weeks, while Gaborit et al. 2021 used novel multiple cardiac imaging modalities combining 1H-MRS and 31P-MRS, and the follow-up occurred after 12 weeks [18].

In other research focusing on SGT2-i, we found three clinical trials in paired studies without control groups: Yagi S et al. [20], in which canagliflozin treatment reduced EAT by 20.34% in 24 weeks; Bouchi R et al. [22], in which luseogliflozin reduced EAT by 5.13% in 12 weeks; and Fukuda T et al. [21], in which ipragliflozin reduced EAT by 12.75% in 12 weeks. After these last studies, the BMI and HbA1c were reduced, but VAT levels were reduced in only two of the three studies. The reduction in EAT was higher than we expected. The precise mechanisms underlying these results are unknown. However, EAT has a higher rate of fatty acid intake and secretin than VAT and functions as a local energy source during times of energy demand. The high metabolic turnover might explain why EAT was more sensitive to SGLT2-i compared to VAT [38].

For decades, clinical decisions about the treatment of T2D have been based on glycemic control. This has changed due to trials demonstrating atherosclerotic cardiovascular disease and chronic kidney disease benefits independent of the glucose-lowering potential of medications [39]. The SGLT2 inhibitor has been recognized as one of the front-line treatment drugs by the new guidelines for the management of T2D [40,41]. As regard to mechanism(s) that might regulate a significant reduction in major cardiovascular events, SGLT2-i may mediate partly through metabolic effects and output volume changes. SGLT2-i prevents the kidneys’ reuptake of glucose and sodium from the proximal convoluted tubule. These drugs lead to an average decrease in HbA1c of 0.6–1.2%, a weight loss of 1.5–2.5 kg, and reductions in SBP of 2.5–4.5 mm Hg [42,43]. Similarly, we found a reduction in triglyceride levels and an incremental increase in HDL levels. These results have also been found in a recent meta-analysis of 48 RCT with SGLT-i [44].

In this work we found no differences in weight, partly due to the fact that in the studies of Sato T et al., the overweight population was studied, whereas in the Gaborit B et al. 2021 study, the population with grade I obesity was included. This could explain the lack of significant difference between the groups. It is important to note that EAT possesses characteristics in relation to the expansion of fat and BMI. The expansion of EAT is different from that of visceral and subcutaneous adipose tissue. Fat depot expansion for visceral and subcutaneous adipose tissue is dependent on adipocyte hypertrophy; however, EAT is dependent on hyperplasia [45]. Therefore, it is assumed that epicardial fat depots respond differently to lifestyle. This is supported by findings that exercise specifically reduces visceral fat depots and EAT volume, even in the absence of weight loss [46]. Moreover, SGLT2-i has been shown to reduce ectopic fat accumulation without changing body weight [47].

EAT has protective properties as a buffer in the coronary arteries and myocardium against lipotoxicity and glucotoxicity in normal conditions. Therefore, the main mechanisms by which SGLT2-i reduce the volume and function of EAT would be glycemic improvement, reduction in lipid values, and weight loss [48]. In addition, the effect of these drugs on inflammatory profile, with a lower production of inflammatory cytokines and a higher production of anti-inflammatory adipokines, may also be a factor in cardiovascular benefit [49,50].

This meta-analysis presented several limitations. Firstly, the statistical heterogeneity was high. However, the results were robust when performing the sensitivity analysis. Secondly, the analysis included only trial-level data without the individual data. Thirdly, our analysis included observational studies. Consequently, the presence of biases and confounding factors was greater than we expected. Finally, as randomized clinical trials on this topic have not been performed yet, relatively few studies were included in our analysis. However, the best evidence available to date was analyzed.

## 5. Conclusions

Our data suggest that the use of SGLT2-i results in a significant reduction in EAT volume in a population with T2D. The results of our research may support the theory that EAT represents a measurable cardiovascular risk factor and a potential modifiable therapeutic target for SGLT2 inhibitors. However, due to the limitations of our research related to the clinical and methodological heterogeneity of the small number of included studies, the true role of EAT in the cardiovascular benefit observed due to the new antidiabetic drugs requires clarification in future studies.

## Figures and Tables

**Table 1:** Characteristics of the included studies.

Authors	Type of Study	TA	TC	Follow-Up	Tool	EAT (cm^3^)	BMI (kg/m^2^)	HbA1c (%)	N
Iacobellis G 2020	RCT	DAPA	MET	24 w	ECHO	8.5 *	36.6	6.8	100
Hiruma S 2021	RCT	EMPA	SITA	12 w	MRI	46.8 *	28.6	7.1	44
Sato T 2018	SAS	DAPA	UC	24 w	CT	98.6	26.6	7.2	40
Gaborit B 2021	RCT	EMPA	PL	12 w	MRI	106.9	32.6	8.1	51
Sato T 2020	SAS	DAPA	UC	24 w	CT	97.8	26.2	7.1	35
Fukuda T 2017	SAS	IPRA	N/A	12 w	CT	102	22.6	7.2	9
Bouchi R 2017	SAS	LUSEO	N/A	12 w	CT	117	28.7	7.5	19
Yagi S 2017	SAS	CANA	N/A	24 w	ECHO	9.3 *	27	7.1	13

CANA = canagliflozin, DAPA = dapagliflozin, ECHO = echocardiography, EAT = epicardial adipose tissue, EMPA = empagliflozin, IPRA = ipragliflozin, LUSEO = luseogliflozin, MRI = magnetic resonance imaging, PL = placebo RCT = randomized controlled trial, SITA = sitagliptin, SAS = single-arm study, TA = treatment active, TC = treatment control. * EAT thickness is a parameter of surface that is used in qualitative analysis (mm or mm^2^).

**Table 2:** Quality assessment (NOS).

Study	Selection	Comparability	Outcome
Sato T (2018)			
Gaborit B (2021)			
Sato T (2020)			

## Data Availability

Not applicable.
